# Pathogenicity of Shiga Toxin Type 2e *Escherichia coli* in Pig Colibacillosis

**DOI:** 10.3389/fvets.2020.545818

**Published:** 2020-09-18

**Authors:** Valentina Baldo, Cristian Salogni, Stefano Giovannini, Mario D'Incau, Maria Beatrice Boniotti, Laura Birbes, Alessandra Pitozzi, Nicoletta Formenti, Andrea Grassi, Paolo Pasquali, Giovanni Loris Alborali

**Affiliations:** ^1^Istituto Zooprofilattico Sperimentale della Lombardia e dell'Emilia Romagna “Bruno Ubertini”, Brescia, Italy; ^2^Department of Veterinary Public Health and Food Safety, Istituto Superiore di Sanità, Rome, Italy

**Keywords:** edema disease, healthy pigs, virulence factors, antimicrobial resistance, ESBL genes

## Abstract

Shiga toxin type 2e (Stx2e) *Escherichia coli* is the causative factor of diarrhea and edema in swine. The aims of this study were to determine the prevalence of Stx2e-producing *E. coli* isolates and to characterize isolates from clinical cases of pig colibacillosis and healthy swine. During the 11 years of the study (2006–2017), a total of 233 Stx2e-producing isolates were detected−230 out of 2,060 (11.16%) *E. coli* isolated from diseased pigs and 3 out of 171 (1.75%) from healthy swine. Stx2e-producing isolates were indeed more present in clinical colibacillosis cases than in healthy pigs (*p* = 0.0002). The predominant serogroup was O139 (79.82%) and the most common fimbrial factor present in these isolates was F18 (177 isolates), followed by F6 (5 isolates). The enterotoxins LTI, STa, and STb were detected in 10.43, 41.73, and 48.26% of the isolates, respectively. The predominant virotypes F18-Stx2e and -STa-STb-Stx2e were similarly present in weaners (33.33 and 35.52%) and finishers (38.30 and 25.53%). Among isolates from diseased pigs, O139 and F18 were the more frequently identified serogroup and virulence factor, respectively. Of the tested 230 Stx2e-producing isolates isolated from diseased pigs, 29 (12.60%) harbored genes encoding ESBL, particularly TEM (79.30%), CTX-M1 (17.20%), and CMY-2 (3.40%). Antimicrobial resistance to tetracycline was the most common characteristic (98.25%), followed by ampicillin (93.91%), cephalotin (90.43%) and trimethoprim/sulfamethoxazole (82.17%). Our results showed that Stx2e-producing *E. coli* were more frequently associated with clinical forms of colibacillosis, with minimal probability to isolate these isolates from healthy pigs.

## Introduction

*Escherichia coli* is a facultative anaerobe (1) and, although an opportunistic pathogen commonly found in the intestinal tracts of vertebrates (2), has the potential to cause seriously pathogenic colibacillosis in humans and animals, when it harbors specific virulence genes (3). Indeed, even if most *E. coli* are harmless, some of them are able to cause gastroenteric/enteric or systemic diseases in vertebrates (4, 5). According to Barbau-Piednoir et al. (6), pathogenic *E. coli* are divided in diarrhoeagenic *E. coli* (DEC) and extraintestinal *E. coli* (ExPEC) and, based on the type of virulence factor present and on the host clinical symptoms, these groups can be further grouped into pathotypes. Uropathogenic (UPEC) and neonatal meningitidis (NMEC) *E. coli* belong to the ExPEC while the DEC group consists of eight pathotypes: shigatoxigenic (STEC) [including the enterohemorrhagic (EHEC)], enteropathogenic (EPEC), enterotoxigenic (ETEC), enteroinvasive (EIEC), enteroaggregative (EAggEC), diffusively adherent (DAEC), adherent invasive (AIEC), and the recently described enteroaggregative shigatoxigenic (EAggSTEC) *E. coli* (6).

Focusing on intensive pig farming, colibacillosis is a major threat due to severe economic losses, consequences of high morbidity, increased mortality rates, stunted or decreased growth, elevated health management costs and elevated costs of pharmacological treatments (7, 8). Among pathogenic *E. coli*, ETEC, and STEC isolates are the main agents in swine causing post-weaning diarrhea (PWD) and edema disease (ED), respectively (9). A common feature of these two pathotypes is the expression of specific fimbrial adhesins that allow the bacterial binding at the mucosal surface of the porcine small intestine, contrasting intestinal peristalsis (5). These fimbriae, indicated with the letter F, are numbered progressively. In swine, isolates typically display specific types of fimbriae, including F4 (K88), F5 (K99), F6 (P987), F18, and F41 (10). The most commonly reported fimbrial adhesins are of the F4 and F18 types, both with different antigenic variants: three for F4 (ab, ac, and ad), with F4ac being the most prevalent, and two main variants for F18 (F18ab is associated with ED and F18ac with PWD (11)). Other associated fimbriae of lower prevalence include F5, F6, and F41, whose number of active receptors on the intestinal epithelial cells decreases with the age of the host (5, 10). In addition to these colonization factors, the pathogenic attitude of ETEC and STEC is also mediated by the ability to produce enterotoxins and/or Shiga-toxins (12), respectively. The effect of both toxins on the digestive system leads to rapid intestinal fluid hypersecretion with consequent sudden onset of osmotic watery diarrhea that frequently results in severe dehydration and circulatory shock (13). The heat-labile enterotoxin LT and the heat-stable enterotoxins STa and STb are the best-known toxins (8). Epithelial adherence of these toxins is predominantly facilitated by adhesive fimbriae, thus according to Renzhammer et al. (8), the detection of at least one enterotoxin gene, together with one gene coding for fimbriae (F4, F5, F6, F18, and F41) in a single *E. coli* is defined as an essential criterion for the classification of porcine ETEC. On the other hand, STEC (Shiga toxin-producing *E. coli*) isolates produce the Shiga-toxins that are classified as type 1 (Stx1: Shiga-toxin 1 type) with subtypes a, c, d, and as type 2 (Stx2: Shiga-toxin 2 type) with subtypes a, b, c, d, e, f and g. Stx2 variant “e,” also called edemigenic toxin, is the causative agent of the severe ED in pigs (9). The ability of STEC to cause disease is related to the production of one or more Shiga-like toxins, which inhibits the protein synthesis of host cells, thus leading to cell death (14). Furthermore, some isolates harbor both the Stx2e and enterotoxin genes, being able to cause symptoms of edema disease and diarrhea in the same animal (STEC/ETEC) (15). Therefore, the fact that ED is almost the most pathogenic among pig colibacillosis, together with the attitude of STEC in general to be important food-borne pathogens (7), representing a serious zoonotic risk with swine playing an important role as a carrier (13), highlights the need to investigate the presence and the characteristics of these pathogenic isolates in pigs.

In order to provide further insights into patterns associated with virulent and non-virulent phenotypes of Stx2e, the present study aims to determine the prevalence, the biomolecular and antimicrobial resistance patterns of Stx2e-producing *E. coli* isolates isolated from cases of pig colibacillosis and from healthy swine.

## Materials and Methods

### Sample Collection

The presence of Stx2e-producing *E. coli* isolates was investigated in 2060 cases of colibacillosis, from January 2006 to December 2017, as part of the routine activity of the Diagnostic Section of IZSLER in Brescia, Italy The presence of Stx2e-producing *E. coli* isolates was investigated in 2060 cases of colibacillosis, from January 2006 to December 2017, as part of the routine activity of the Diagnostic Section of IZSLER in Brescia, Italy. In particular, a total of 1,337 *E. coli* were isolated from small intestinal contents sampled during the necropsy of pigs (Table 1) conferred to our Department for further diagnostic investigations. At the macroscopic examination these pigs showed yellowish, gray, or slightly pink watery diarrhea with a characteristic smell; the small intestine were dilatated, slightly edematous and hyperaemic with enlarged and hyperaemic mesenteric lymph nodes. Moreover, edema disease occurred in 24 of these cases and were characterized by edema in various sites, e.g., eye lids, nose bridge and forehead, stomach wall, and the mesentery of the colon, as main finds of edema disease as Franke et al. (16) had previously reported. In addition, an overall 723 *E. coli* were isolated from fecal samples/fecal swabs of living pigs (Table 1) with diarrhea. Carcasses and fecal samples were conferred to our Department from a total of 670 farms (no more than five samples from the same farm during the whole study period). All of these farms make a regular use of antibiotics.

**Table 1:** Sampling details for each pig category (suckling, weaner, and finisher).

	**Suckling (21–25 days old)**		**Weaner (60–70 days old)**		**Finisher (6–7 months old)**	
**Study year**	**Fecal samples**	**Small intestinal contents**	**Fecal samples**	**Small intestinal contents**	**Fecal samples**	**Small intestinal contents**
2006	19	37	22	59	19	24
2007	18	34	22	51	18	17
2008	15	29	19	53	16	18
2009	20	39	24	77	22	18
2010	18	35	24	57	18	18
2011	24	46	25	64	24	22
2012	18	35	20	50	19	16
2013	19	37	24	57	21	17
2014	20	39	20	56	26	19
2015	19	36	24	52	21	23
2016	16	32	16	50	18	15
2017	18	35	18	55	19	15

The presence of Stx2e-producing *E. coli* isolates was also investigated in 171 living pigs−29 weaners and 142 finishers—without clinical any signs of colibacillosis sampled from 18 distinct farms during 2016. Farmers were voluntarily involved in the study and who agreed to participate allowed us to collect feces and/or fecal swabs from their swine.

This study was carried out as a part of the routine activity of Diagnostic Section of Istituto Zooprofilattico Sperimentale della Lombardia e dell'Emilia Romagna (IZSLER), thus the scientific protocol did not require an additional approval of the Ethical Committee for Animal Experimentation of IZSLER.

### Identification of *E. coli*

The isolation procedure was consistent during the whole study period (2006–2017). The samples, processed within 24 h after the collection, were cultured on MacConkey agar plates and blood agar plates (Oxoid, Italy) and incubated aerobically for 18 ± 2 h at 37 ± 2°C. After an overnight incubation, suspicious *E. coli* colonies were identified by morphology (pink on MacConkey and/or with hemolysis on blood agar plates) and Gram staining. For each case/animal, one suspected colony with presumptive biochemical properties (lactose and indole positive; H_2_S, oxidase, and urease negative) was subcultured on BHI (Brain Heart Infusion) agar slant (Oxoid, Italy) while its identity was confirmed by the biochemical method, API 20E (bioMérieux, France).

All the isolates were preserved in medium containing tryptone soy broth (TSB) with 20% glycerol at −80°C.

### Serogrouping of the Isolates

The serogrouping of the Stx2e-producing *E. coli* isolated from clinical cases of colibacillosis was based on somatic O-antigens since, among them, there are the most frequent and pathogenic for humans and animals (17). The analyses was carried out using agglutinating antisera in microplate according to Guinée et al. (18) and modified by Blanco et al. (19). The available antisera were against 30 serogroups from O1 to O157 (O1, O2, O5, O8, O9, O15, O18, O20, O22, O26, O45, O49, O55, O64, O78, O86, O88, O101, O103, O111, O113, O118, O128, O138, O139, O141, O147, O149, O153, O157) (Oxoid, Italy) were tested. Briefly, in 96-well plates, 100 μl of diluted antiserum and 100 μl of O-antigens suspensions prepared by heating bacterial suspensions for 1 h at 100°C were mixed into each well and incubated at 37 ± 2°C for 18 ± 2 h. A positive reaction was confirmed by agglutination in the diluted antiserum. Isolates that did not react with any of the O-antisera examined were classified as O-antisera untypeable (ND).

### Molecular Characterization by Multiplex Real-Time PCR

*Escherichia coli* isolated from both clinical cases and healthy pigs were screened by multiplex PCR for the presence of the major virulence genes of porcine pathogenic *E. coli*, including genes for 5 different adhesins (K88, K99, F41, 987P, and F18) and 4 different toxins (LT, STaP, STb, and Stx2e) following the method according to Casey and Bosworth (20). Briefly, DNA was obtained from each *E. coli* isolate (one colony) by hot lysis procedure in which the sample were harvested by centrifugation (12.000 × g for 5 min), and washed three times in distilled water, boiled at 97.5 ± 2.5°C for 10 min and immediately cooled on ice for 2 min. After centrifugation, the extracted DNA was subjected to multiplex PCR for screening of virulence factors (VFs) using specific primers (Table 2). According to Casey and Bosworth (20), the PCR reaction mixtures contained 18 primers at a concentration of 0.5 μmol each, 0.2 mmol deoxyribonucleotide triphosphate mix, 1 X reaction buffer, 5 mmol MgCl_2_, and 2.5 units of Taq polymerase in a final volume of 20 μl. These amplification conditions were followed: an initial denaturation at 94°C for 10 min, followed by 30 cycles of denaturation for 30 s at 94°C, annealing at 55°C for 45 s, and extension for 1.5 min at 72°C. The extension time was increased by 3 s each cycle, and the final extension was 10 min at 72°C. The amplification products were then separated and detected by electrophoresis using 4% agarose gels at 75–100 V for 1.5–2 h (20).

**Table 2:** PCR primers used for the detection of *E. coli* virulence factors (VFs) and extended-spectrum β-lactamases (ESBL).

	**Gene**	**Primers sequence**	**5^**′**^-3^**′**^**	**Product size**
***E. coli*** **Virulence Factors (VFs)**	K99	F5 F	AATACTTGTTCAGGGAGAAA	230
		F5 R	AACTTTGTGGTTAACTTCCT	
	F18	F18 F	TGGTAACGTATCAGCAACTA	313
		F18 R	ACTTACAGTGCTATTCGACG	
	987P	987P F	AAGTTACTGCCAGTCTATGC	409
		987P R	GTAACTCCACCGTTTGTATC	
	K88	F4 F	GTTGGTACAGGTCTTAATGG	499
		F4 R	GAATCTGTCCGAGAATATCA	
	F41	F41 F	AGTATCTGGTTCAGTGATGG	612
		F41 R	CCACTATAAGAGGTTGAAGC	
	LTb subunit	LT F	GGCGTTACTATCCTCTCTAT	272
		LT R	TGGTCTCGGTCAGATATGT	
	STb	STb F	TGCCTATGCATCTACACAAT	113
		STb R	CTCCAGCAGTACCATCTCTA	
	STa	STaP F	CAACTGAATCACTTGACTCTT	158
		STaP R	TTAATAACATCCAGCACAGG	
	Stx2e	Stx2e F	AATAGTATACGGACAGCGAT	733
		Stx2e R	TCTGACATTCTGGTTGACTC	
**Extended-spectrum b-lactamases**	CTX-M1	Group 1	AAAAATCACTGCGCCAGTTC	415
			AGCTTATTCATCGCCACGTT	
	CTX-M2	Group 2	CGACGCTACCCCTGCTATT	552
			CCAGCGTCAGATTTTTCAGG	
	CTX-M9	Group 9	CAAAGAGAGTGCAACGGATG	205
			ATTGGAAAGCGTTCATCACC	
	CTX-M8/25	Groups 8	TCGCGTTAAGCGGATGATGC	666
			AACCCACGATGTGGGTAGC	
		Groups 25	GCACGATGACATTCGGG	327
			AACCCACGATGTGGGTAGC	
	SHV	OS5	TTATCTCCCTGTTAGCCACC	790
		OS6	GATTTGCTGATTTCGCTCGG	
	AmpC-gene CMY	CMY-2-F	ATGATGAAAAAATCGTTATGCTGC	1117
		CMY-2-R	GCTTTTCAAGAATGCGCCAGG	
	TEM gene	TEM F	ATAAAATTCTTGAAGAC	1200
		TEM R	TTACCAATGCTTAATCA	

### Antimicrobial Susceptibility Testing

The susceptibility of Stx2e-producing isolates to a panel of antimicrobials was tested using the disc diffusion method following the procedures of the Clinical and Laboratory Standards Institute (Clinical and Laboratory Standards Institute (CLSI) (21–26). Briefly, the isolates were inoculated in trypticase soy broth (TSB) and then plated on Mueller-Hinton agar. The following 11 commercially available antibiotic discs were used: ampicillin (AMP: 10 μg), amoxicillin/clavulanic acid (AMC: 30 μg), cephalothin (KF: 30 μg), ceftiofur (EFT: 30 μg), enrofloxacin (ENR: 5 μg), florfenicol (FFC: 30 μg), flumequine (FQ: 30 μg), gentamicin (G: 10 μg), kanamycin (K: 30 μg), tetracycline (TE: 30 μg), trimethoprim/sulfamethoxazole (SXT: 1.25/23.75 μg). The plates were read after incubation in aerobic condition at 37 ± 2°C to 18 ± 2 h. The isolates were classified as resistant, susceptible or intermediate to the antimicrobials tested according to the zone diameter interpretative standard recommendations by CLSI (M45-A; M2-A9; M100-S26; VET08; M100-S29). The isolates with intermediate growth were considered to be resistant (27).

The identification of broad spectrum β-lactamase -producing *E. coli* was performed through a double synergy diagnostic method: after the pre-enrichment with BHI broth supplemented with 1 mg/L cefotaxime and an overnight incubation, a drop of the BHI broth was used to inoculate MacConkey agar supplemented with 1 mg/L cefotaxime (28–30). Positive growths were identified as pink to dark-pink colonies and one of these was selected for further molecular characterization.

### Characterization of β-Lactamase Genes

Detection of the resistance genes present in the Stx2e-producing isolates isolated from diseased pigs was performed using a panel of PCR reactions. A multiplex PCR was used for the identification of the CTX-M group genes whose single or multiple positivity identifies the five main phylogenetic groups—CTX-M1, CTX-M2, CTX-M9 and CTX-M8 and CTX-M25 (31). In addition, single PCR reactions were used for the identification of the SHV gene (32), TEM gene (33), using universal primers as previously described (34–37), and AmpC genes (CMY-2, CMY-4, CMY-6, CMY-7, CMY-12, CMY-13, CMY-14, CMY-18, LAT-3) [(38); Table 2]. All the TEM and SHV PCR amplicons were DNA-sequenced.

### Statistical Analysis

Comparison between groups was assessed by using Fisher's exact test and differences were considered significant when *P* < 0.05.

## Results

### Sample Identification and Molecular Characterization

A total of the 2,060 *E. coli* were isolated from diseased pigs. Of these, 230 (11.16%) were positive for Stx2e-producing isolates (Table 3), β-hemolytic activity was recorded in 215 (93.48%) of the isolates. All the details about the Stx2e-producing E. coli isolated from diseased pigs during each study year are available in Table S1. Three out of 171 (1.75%) *E. coli* isolated from healthy pigs harbored the Stx2e toxin (Table 3). Stx2e-producing isolates were more present in clinical colibacillosis cases than in healthy pigs (*p* = 0.0002).

**Table 3:** Proportion of Stx2e-producing isolates *E. coli* isolated in diseased and healthy weaners and finishers pigs.

	**Stx2e-producing isolates**	
**Age group**	**Diseased pigs**	**Healthy pigs**
Suckling	0/658 (0.00%)	0/0 (0.00%)
Weaner	183/939 (19.50%)	0/29 (0.00%)
Finisher	47/463 (10.15%)	3/142 (2.11%)

### Serogrouping of the Isolates

The serogrouping of the 230 Stx2e-producing *E. coli* isolated from diseased pigs showed that 109 (47.39%) of them were typeable with available O-antisera, whereas 121 (52.61%) isolates could not be assigned to any of the 30 serotypes serogroups tested and defined as O-antisera untypeable (ND). A total of 11 serogroups were identified within the collection: O1 (0.92%, 1/109), O2 (2.75%, 3/109), O6 (0.92%, 1/109), O8 (2.75%, 3/109), O9 (0.92%, 1/109), O128 (1.83%, 2/109), O139 (79.82%, 87/109), O141 (4.59%, 5/109), O147 (2.75%, 3/109), O149 (0.92%, 1/109), O157 (1.83%, 2/109).

### Virulence Factors (VFs) Detected in Stx2e-Producing Isolates

**Figure 1:**
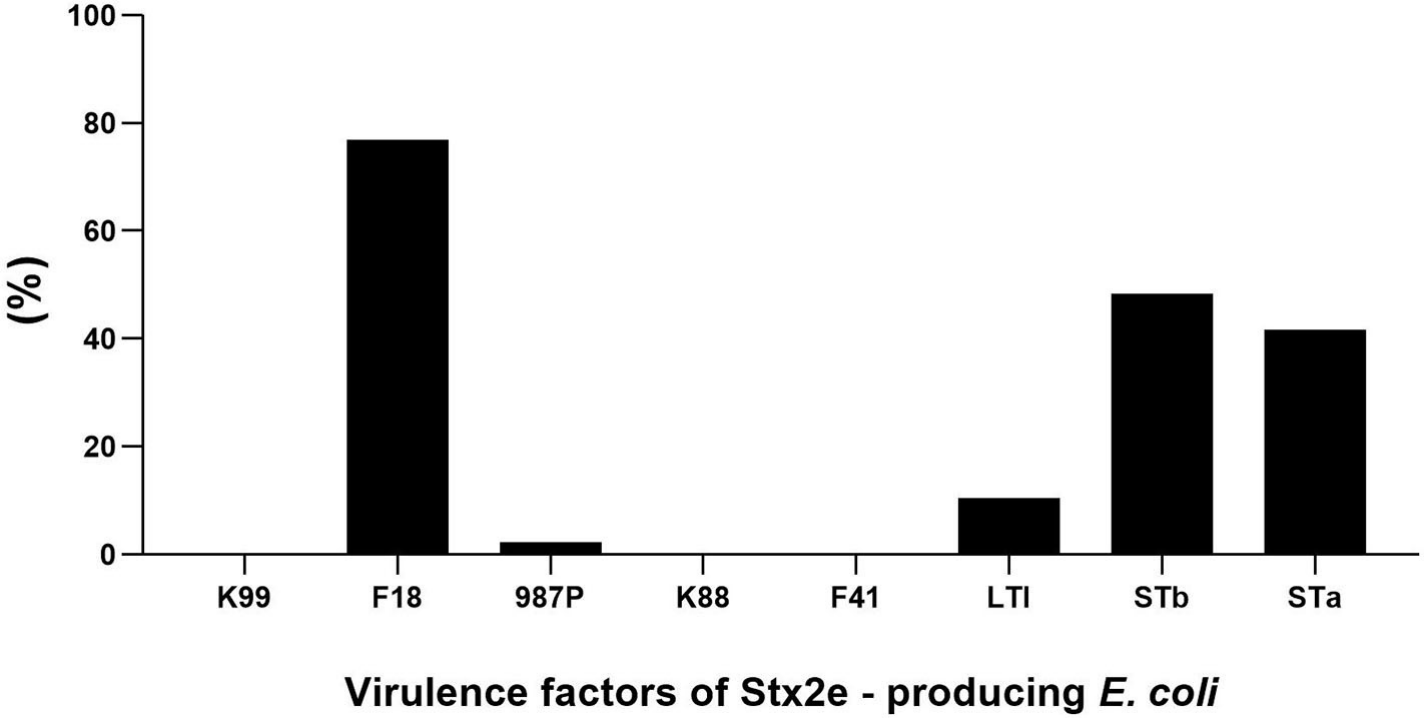
Distribution of fimbrial factors and enterotoxins in the overall analyzed 230 Stx2e-producing isolates.

Fimbrial factors were detected in 182 (79.13%) of the 230 Stx2e-producing isolates isolated from clinical colibacillosis cases. The most common fimbrial factor in the isolates was F18 (177 isolates) followed by 987P (5 isolates) (Figure 1). Differences in the presence of fimbrial factors between weaners and finishers are shown in Table 4. In particular, F18 was more frequently found in weaner than in finishers (*p* < 0.05). The enterotoxin LTI was detected in 24 out of 230 (10.43%) isolates; STa was detected in 96 out of 230 (41.73%) Stx2e-producing isolates; while STb was detected in 111 out of 230 (48.26%) Stx2e-producing isolates recorded (Figure 1). In addition, the prevalence of predominant virotypes, F18-Stx2e and F18-STa-STb-Stx2e, was similarly present in weaners (33.33 and 35.52%) and finishers (38.30 and 25.53%) (Table 4). No virulence factors were detected in the 3 Stx2e-producing *E. coli* isolated from healthy pigs.

**Table 4:** Prevalence of virotypes of Stx2e-producing *E. coli* isolated from weaner and finisher pigs.

**Stx2e—producing *E. coli* virotyping**	**Weaner pigs**	**Finisher pigs**
F18-STa-STb-STx2e	35.52% (65/183)	25.53% (12/47)
F18-STx2e	33.34% (61/183)	38.30% (18/47)
STx2e	6.01% (11/183)	29.79% (14/47)
F18-STb-STx2e	6.01% (11/183)	2.13% (1/47)
LTI-STa-STb-STx2e	4.37% (8/183)	0.00% (0/47)
F18-STa-STx2e	2.73% (5/183)	0.00% (0/47)
LTI-STx2e	2.73% (5/183)	2.13% (1/47)
LTI-STb-STx2e	2.19% (4/183)	0.00% (0/47)
987P-LTI-STb-STx2e	1.64% (3/183)	2.13% (1/47)
STa-STb-STx2e	1.64% (3/183)	0.00% (0/47)
F18-LTI-STb-STx2e	1.09% (2/183)	0.00 (0/47)
987P-STx2e	0.55% (1/183)	0.00% (0/47)
STb-STx2e	0.55% (1/183)	0.00% (0/47)
STa-STx2e	0.55% (1/183)	0.00% (0/47)

### Antimicrobial Susceptibility Testing

**Figure 2:**
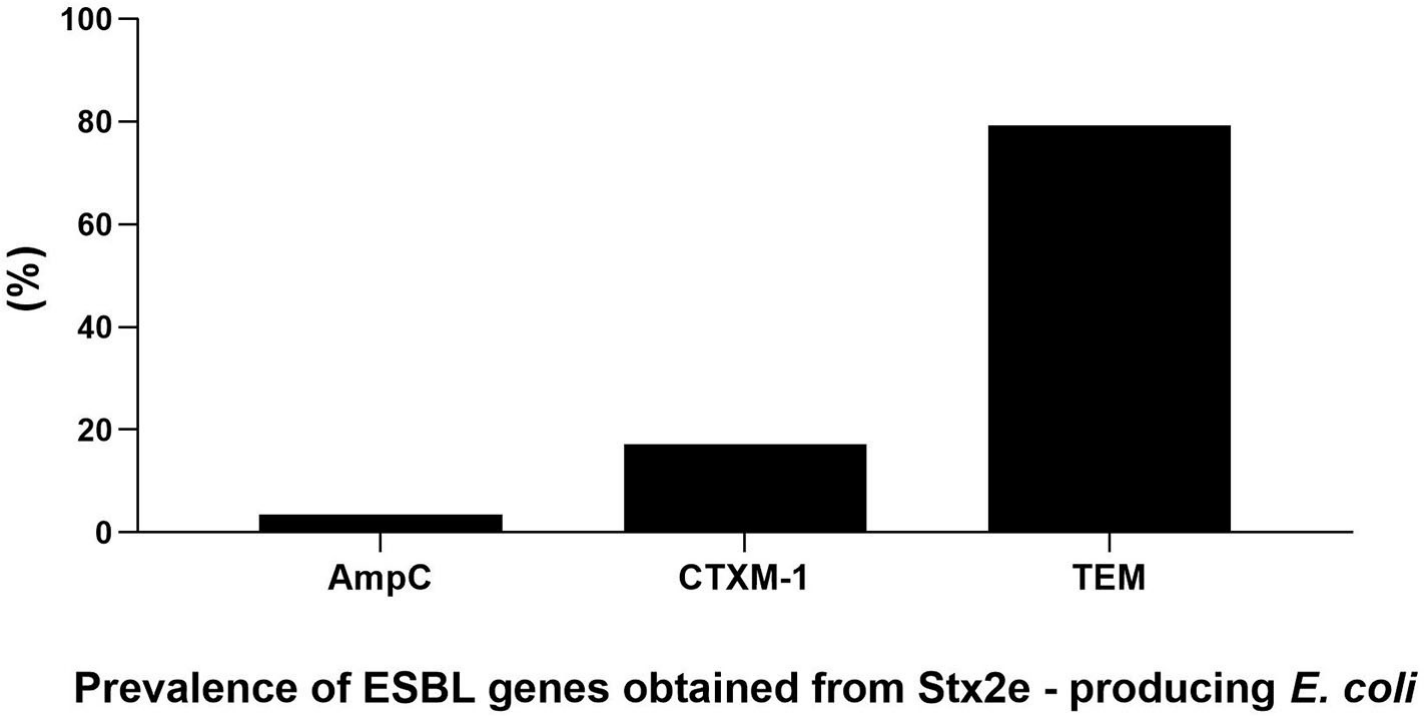
Prevalence and genetic characterization of ESBL genes obtained from the overall 230 Stx2e-producing isolates isolated from diseased pigs.

The antimicrobials resistance profile of the Stx2e-producing *E. coli* isolated from diseased pigs was determined against 11 antibiotics (Table 5). The whole panel of antimicrobials had been applied on 189 Stx2e-producing isolates, for which frequency of multi-drug resistance (MDR) was calculated (Table 5), since not all the considered antibiotics have been available during all the study period. All these isolates were resistant to at least one antibiotic (Tables 5, 6). Resistance to tetracycline had the highest prevalence with a rate of 98.25%; ampicillin resistance showed a rate of 93.91% and resistance to cephalotin a rate of 90.43% (Table 5). Other common resistances possessed by the Stx2e-producing isolates detected in clinical colibacillosis cases were to trimethoprim/sulfamethoxazole, kanamycin, gentamicin, followed by resistance to amoxicillin/clavulanic acid, flumequine, ceftiofur, florfenicol, and enrofloxacin (Table 5). The overall possible multi-drug resistant profiles of the Stx2e-producing E. coli for which the whole panel of antimicrobials has been applied are available in Table S2. Out of the 230 Stx2e-producing isolates recorded from diseased pigs, 29 (12.60%) harbored genes encoding ESBL (Figure 2). The ESBL genes identified were TEM (79.30%), CTX-M1 (17.20%), and CMY-2 (3.40%) (Figure 2). From the total of 23 Stx2e-producing *E. coli* TEM-positive, overall 21 of them were available for sequencing and showed TEM-1 as result. PCR products were sequenced and deposited in NCBI GenBank with accession numbers from MT789713 to MT789733.

**Table 5:** List of antimicrobials included in the Kirby-Bauer disc diffusion method, Number (N°) of resistant and tested Stx2e-producing isolates and the percentages of antimicrobial resistance identified in this study.

**Antimicrobial resistance of Stx2e—producing isolates**			
**Antimicrobials**	**N°** **of resistant isolates**	**N°** **of tested isolates**	**Percentage of antimicrobial resistance**
ENR	80	222	36.04%
EFT	94	229	41.05%
FQ	102	230	44.35%
FFC	93	201	46.27%
AMC	108	227	47.58%
G	132	230	57.39%
K	152	230	66.09%
SXT	189	230	82.17%
KF	208	230	90.43%
AMP	216	230	93.91%
TE	225	229	98.25%

**Table 6:** Distribution of concurrent resistance in Stx2e-producing *E. coli* isolated from diseased pigs.

**Count of concurrent antimicrobial resistance**	**Percentage of concurrent antimicrobial resistance**
1	1.59% (3/189)
2	0.53% (1/189)
3	1.59% (3/189)
4	10.05% (19/189)
5	12.70% (24/189)
6	13.76% (26/189)
7	16.40% (31/189)
8	15.87% (30/189)
9	9.52% (18/189)
10	12.70% (24/189)
11	5.29% (10/189)

## Discussion

In the present study, Stx2e-producing *E. coli* were isolated and characterized from diseased and healthy weaner and finisher pigs, in light of the considerably different distribution and frequency of serogroups and virotypes between production stages (10, 19). A total of 230 isolates obtained from clinical forms of colibacillosis showed the Shiga-toxin variant 2 (Stx2e). Only three cases of Stx2e-producing isolates were isolated from healthy pigs. Thus, the Stx2e variant appears to be more frequently associated with clinical colibacillosis. The O139 serogroup and the virulence factor F18 were isolated in Stx2e-producing isolates from diseased pigs, suggesting that these characteristics are associated with a virulent phenotype.

The presence of Stx2e-producing isolates in cases of colibacillosis in pigs is well-documented although our study recorded higher prevalence than previously reported. For instance, Chen et al. (39) pointed out that Stx2e-producing isolates were isolated in 6.1% of the 250 *E. coli* isolates collected from pigs with diarrhea. A similar prevalence was reported by Brand et al. (40), who found 3.36% of Stx2e-producers in *E. coli* isolates from 115 pigs with diarrhea. Moreover, the presence of Stx2e-producing *E. coli* in healthy pigs has not been well-investigated although these isolates were normally isolated from healthy swine (41). Meng et al. found the presence of these subtype isolates in 25.42% of healthy swine in slaughterhouses (13). Similar result emerged in a recent study by Arancia et al., which showed a prevalence of 25.8% for Stx2e subtype isolates (52.1%) in the caecal contents of slaughtered pigs (41). Compared to these studies, we observed a lower prevalence of Stx2e subtype isolates in healthy pigs. This finding could be ascribed to possible differences in the screening methods employed. In the work by Meng et al. (13), the reported rate of 25.42% of STEC in healthy pigs was obtained by PCR screening; however, only 6.18% of the swine samples yielded STEC isolates by microbiological culture. Moreover, the difference in reported prevalence of isolation could be due to the anatomic sites of sampling. Indeed, the rate of isolation of STEC in fecal samples was considerably lower than the rate from colon or the small intestine (13). As this regards, the fact of having sampled feces/fecal swabs of healthy pigs and the known presence in animal feces of numerous materials (e.g., complex polysaccharides, bilirubin, and bile salts) that are inhibitors of PCR (42) should be considered in the interpretation of results, although the reliability of both the used DNA extraction and PCR protocols.

We found that the vast majority of the isolated Stx2e-producing isolates belong to the O139 serogroup. Interestingly, available studies have mainly focused on the distribution of O157, without consideration for other serogroups. Milnes et al. (43) reported the detection of 0.3% (6/2,000) of VTEC O157 isolates from swine fecal samples at slaughter facilities. Similarly, Lenahan et al. (44) recorded a prevalence of STEC O157:H7 of 0.6% (3/480) in fecal samples from swine at slaughterhouses, supporting the report by Bonardi et al. (45) who isolated a low proportion (0.7%, 1/150) of O157-STEC isolates from fecal samples at slaughter facilities. Several European studies have reported STEC prevalence in swine population and most focused on STEC serogroup O157 (39, 46). However, in the present study, O157 was isolated in only one case out of the 230 investigated. Further, Friedrich et al. (47) showed that pigs with edema disease in post-weaning and young finishing pigs caused by STEC presented with serogroups O8, O138, O139, O141, and O147, similar to findings on Stx2e isolates reported by Fratamico et al. (14). Overall, these data suggest that, although O157 seemed an important serogroup from a public health perspective, other serogroups can have a significant impact on animal health, in certain types of animals (7).

In our study, most of the Stx2e-producing isolates harbored F18 adhesin factor, with a lower percentage of samples possessing 987P fimbriae; K88, K99, and F41 were not observed. These findings are not surprising, as the gene encoding F18 fimbriae is among the most important ones associated with colibacillosis and edema disease (10). Likely the fact that all the Stx2e-producing *E. coli* were detected from diseased animals with diarrhea supports an effect of the co-presence of these two virulence factors (Stx2e toxin and F18 adhesin) in causing this symptom, although even Shiga toxigenic *E. coli* are reported to cause diarrhea in young pigs (48–50). F18 fimbriae are absent in most human-derived STEC, but are essential for adherence to swine epithelial cells (13). Chen et al. (39) reported that 50.2% *E. coli* isolates detected from pigs with post-weaning diarrhea carried one or more fimbrial factors. In the present study, data from the multiplex PCR on Stx2e-producing isolates showed that STa and STb positive isolates were more prevalent than LTI. This fact, in light of the attitude of these enterotoxins in causing diarrhea and dehydration in pigs (51), may be related to the diarrheic syndrome recorded in the most of the analyzed subjects. In addition, this finding agrees with previous studies by Chen et al. (39) that reported 60.5% STa in isolates from pigs with post-weaning diarrhea, and Toledo et al. (52), which observed STa (30%), STb (17%), Stx2e (6%), and LTI (5%) as the most common enterotoxins in suckling and weaning pigs.

The presence of extended-spectrum β-lactamase producing *Escherichia coli* (ESBL) in humans and animals is a major global public health concern (53). In the present study, 29 out of the 230 Stx2e–producing isolates (12.60%) isolated from diseased pigs possessed ESBL genes. Although STEC isolates are not deemed a reservoir of ESBL, in recent reports have indicated an association between STEC isolates and ESBL genes, Mandakini et al. (53) reported on 25.29% isolates phenotypically confirmed as ESBL producers. We found that the predominant genes in our ESBL isolates were resistance to TEM (79.30%), CTX-M1 (17.20%), and CMY-2 (3.40%), in contrast to Jones et al. (54) who pointed out that CTX-M resistant genes were detected in 85% of isolates while TEM resistance was found in 6.12%. However, although the phenotypical test carried out in our study demonstrated the resistance of these isolates to cefotaxime, the fact that sequencing analyses showed the presence of TEM-1 Stx2e–producing *E. coli* cannot exclude the possibility that these isolates could have any other susceptibility to other cephalosporins (55).

Antimicrobial resistance (AMR) has become a major public health concern because bacteria causing infectious diseases are becoming less susceptible to antibiotic treatment (56, 57). However, antibiotic resistance can also arise in opportunistic bacteria, such as *E. coli*, as a result of different mechanisms (56). Currently, the presence and the dissemination of AMR among the genus *Escherichia* spp. represents an emerging problem (56, 57). In the present study, Stx2e-producing isolates isolated from farmed pigs showed high levels of resistance to various antimicrobial agents. Although the use of the Minimum Inhibitory Concentration (MIC) test is currently suggested for antimicrobial resistance detection, we employed the Kirby-Bauer disc-diffusion method because specimen from 11 years of study were included, with results dating back to 2006 when MIC was not routinely performed. The highest percentages of antimicrobial resistance recorded for Stx2e-producing strains isolated in this study were registered for tetracycline (98.25%), ampicillin (93.91%), cefalotin (90.43%), and trimethoprim/sulfamethoxazole (82.17%). These findings appeared not related to a specific correlation between Stx2e toxins and phenotypical antimicrobial resistance but these high percentages could be ascribed to the wide use of these antibiotics in the past for treating pig respiratory and enteric bacterial diseases (27). Moreover, the high percentage of isolates resistant to antibiotics commonly used to treat diarrhea in pigs emphasizes the importance of avoiding unnecessary use of antibiotics. The observed high level of resistance to tetracycline and ampicillin is probably a direct consequence of the intense use of these antibiotics in veterinary medicine. In addition, the horizontal transfer of these resistant genes should be considered together with their potential acquisition from a contaminated external environment (58, 59). We found that, besides the high rate of resistance to most of the tested antimicrobials, the majority of Stx2e-producing isolates isolated from pigs also exhibited multidrug resistance. This data agrees with results from a study by Brand et al. (40) that found a high level of AMR, in particular to tetracycline (50%), sulfamethoxazole (49%), trimethoprim (34%), and ampicillin (26%) in *E. coli* isolates from pigs. These results and the increasing awareness on drug-resistant *E. coli* strains, related to the prophylactic use of antibiotics on healthy piglets or as food additive (60), highlight the need to develop a vaccine against Stx2e-producing isolates as an useful method alternatively to antimicrobials (61). In addition, an effective vaccine program is desirable in order to prime immunity able to confirm protection to the effect of toxins (60).

In conclusion, the present study contributes to strengthening available information on the virulence factors of Stx2e-producing isolates in diseased and healthy pigs. In addition, this survey sheds new light on potential pathogenic characteristics of Stx2e-producing isolates and points out the need for a rational management of antibiotics use and effective vaccination programs in swine farms in order to minimize the impact of these antimicrobial resistant pathogenic Stx2e-producing *E. coli* isolates on animal health.

## Data Availability Statement

The raw data supporting the conclusions of this article will be made available by the authors, without undue reservation.

## Ethics Statement

Ethical review and approval was not required for the animal study because this study was carried out as a part of the routine activity of Diagnostic Section of Istituto Zooprofilattico Sperimentale della Lombardia e dell'Emilia Romagna (IZSLER), thus the scientific protocol did not require an additional approval of the Ethical Committee for Animal Experimentation of IZSLER.

## Author Contributions

VB, CS, SG, PP, and GA contributed to the conception and design of the study. VB, LB, AP, MD'I, MB, and AG performed the experiments. VB and LB organized the database. VB performed the statistical analysis and wrote the first draft of the manuscript. NF and MB wrote sections of the manuscript. All authors contributed to manuscript revision, read, and approved the submitted version.

## Conflict of Interest

The authors declare that the research was conducted in the absence of any commercial or financial relationships that could be construed as a potential conflict of interest.
